# Digital Health Interventions in Depression Care—A Survey on Acceptance from the Perspective of Patients, Their Relatives and Health Professionals

**DOI:** 10.3390/healthcare10102019

**Published:** 2022-10-13

**Authors:** Jessica Hafner, Simone Schönfeld, Pinar Tokgöz, Katharina Choroschun, Arndt Schlubach, Christoph Dockweiler

**Affiliations:** 1 Department of Digital Health Sciences and Biomedicine, School of Life Sciences, University of Siegen, 57068 Siegen, Germany; 2 Institute for Integrative Health Care and Health Promotion (IGVF), Faculty of Health/School of Medicine, Witten/Herdecke University, 58448 Witten, Germany; 3 Department of Company Development and Health Services Research, LWL Kliniken Lippstadt and Warstein, 59556 Lippstadt, Germany; 4 School of Public Health, Bielefeld University, 33615 Bielefeld, Germany

**Keywords:** digital health intervention, mental health, depression, acceptance

## Abstract

Digital health interventions may contribute to closing the treatment gap for depression by reaching large populations at relatively low costs. This article presents the results of a broad, multisided German survey in 2020 on the acceptance and use of digital health interventions in depression care from the perspective of patients, their relatives, and health professionals. A total of 97 patients and relatives and 229 health professionals participated. Survey participants reported openness towards the use of digital health interventions in depression care but little knowledge and experience in the field. Digital health interventions appear to be a promising opportunity for reducing depressive symptoms and shortening waiting time for depression treatment, especially in rural areas. Providing information and technical competencies may increase awareness and knowledge about digital health interventions and the benefits of depression care.

## 1. Introduction

Depression is the most common mental disorder, with an estimated prevalence of 8.3% in Germany in 2019/20. Furthermore, mental health disorders increased tremendously across the globe as a consequence of the COVID-19 pandemic [1]. There is a tendency toward insufficient treatment provision due to access barriers and long waiting times [2], fear of stigmatization, or lack of knowledge concerning treatment options [3]. Digital health interventions (DHIs) could be a solution to overcome existing or potential problems [4,5]. Barriers regarding accessibility due to regional distances as well as limited time resources and availability of licensed psychotherapists especially hinder patients from utilizing necessary treatments [6]. There is convincing evidence that such approaches are effective for depression, show higher effects with increased guidance [7], and even have similar effects when compared with face-to-face treatments [8]. However, DHIs might not be acceptable and suitable for all patients [7,9].

The usage of a DHI depends on its clinical effectiveness as well as its acceptance by patients and health professionals. In Germany, knowledge about the acceptance of such technologies is limited. Technology use has increased with the pandemic and has benefited the acceptance process [10]. In 2020, 77% of psychotherapists reported using video therapy [11]. However, using DHI-like applications with psychoeducational content or self-management tools remain rare in routine care [10,12]. Studies allow only limited conclusions about acceptance since the specific characteristics of depression are not considered adequately [10]. Symptom severity is a substantial barrier to use even among people who are interested in using it [13]. Prior research was collected before the COVID-19 pandemic but considered affected persons in psychiatric care [10,14,15] or health professionals only [16]. Furthermore, those studies refer to hypothetical care scenarios and do not implement and validate DHIs [17]. Additionally, the perspective of relatives and other professions such as social workers, often involved in treatment, is rarely explored. Therefore, this research aims to investigate and describe patients’, relatives’, and health professionals’ perceptions of the acceptance and use of DHI for depression care.

In this study, DHIs are various digital systems that recognize and treat diseases. They support a self-determined and healthy lifestyle and can be used by patients only or together with health professionals. It has been verified that there is no risk in using them. DHI is an umbrella term that includes a wide range of electronic services (e.g., self-monitoring and self-help therapy, video consultation with patients or health professionals, and virtual reality), which can be used, for example, via smartphone, tablet, or PC.

## 2. Materials and Methods

### 2.1. Consideration of Ethical Issues

This online-based survey was conducted in accordance with the Declaration of Helsinki, and the protocol was approved by the Ethics Committee of the University Bielefeld (EUB 2019-041). All subjects gave their informed consent for inclusion before they participated in the study.

### 2.2. Participants

Participants were required to meet the following criteria: (1) All participants had to be 18 years of age or older, (2) patients had to have received an official diagnosis of mild to moderate depression, (3) relatives had to confirm that the affected person received an official diagnosis of mild to moderate depression, (4) health professionals had to have engaged in the care of people with depression and belong to one the following professional groups: Specialist in neurology, specialist in psychiatry and psychotherapy, medical and psychological psychotherapist, health care and nursing staff, social worker, occupational therapist, art therapist, music therapist, or medical assistant. Patients and their relatives were explicitly asked for an official diagnosis of mild to moderate depression. There was a filter question in the survey. Questionnaires were excluded from the analyses if survey participants did not confirm an official diagnosis.

### 2.3. Research Subject and Data Collection Methods

The survey was conducted using the tool *Unipark*. No prior surveys existed on patients’, relatives’, and health professionals´ acceptance of DHI for depression care. Initially, a survey was developed according to the determinants of the Unified Theory of Acceptance and Use of Technology (UTAUT) of Venkatesh et al. (2003), which integrates determinants across eight models to analyze peoples´ intention to use or their actual use of technologies [18]. Venkatesh et al. (2003) utilized the UTAUT model to understand human acceptance behavior across various disciplines. This study utilizes this model, which integrates the following four determinants:

- Performance Expectancy (PE): Refers to the degree to which an individual believes that using the system will help him or her to attain gains.
- Social Influence (SI): Refers to the degree to which an individual perceives that important others believe he or she should use the new system.
- Effort Expectancy (EE): Refers to the degree of ease associated with the use of the system.
- Facilitating Conditions (FC): Refers to the degree to which an individual believes that an organizational and technical infrastructure exists to support the use of the system.

In the UTAUT, PE, EE, SI, and FC are directly associated with behavioral intentions. In addition, behavioral intentions are influenced by gender, age, experience, and voluntariness [14,18].

Differentiated survey questions for patients, their relatives, and health professionals were developed. The questionnaires contained 17 sections with a total of 94 items for health professionals and 80 items for patients and their relatives. For example, the sections included questions about the working field, previous experiences with DHI, advantages and disadvantages of DHI, demographic aspects, PE, SI, EE, and FC. The compilation of items was based on prior literature research and individual interviews. Nineteen problem-centered interviews were conducted with patients with mild to moderate depressive disorders, their relatives, and healthcare professionals in a previous study. The data of the qualitative analysis were compiled through guideline-based interviews and evaluated by structural content analysis according to Kuckartz [19].

The survey questions were presented in the form of four-point Likert scales, yes/no options, and ranking alternatives. Beyond standard response options, the survey allowed free text comments and the “not applicable” alternative. DHI was not expected to be familiar to respondents and was presented in explanatory terms. Cognitive pre-tests (*n* = 8) were conducted to assess the comprehensibility of the items and response options. Further, participants’ demographic characteristics were collected (age, sex, highest professional degree, profession, treatment sector, depression diagnosis, and kind of treatment). In order to reduce inhibition, it was not asked whether the person is a patient or a relative.

Patients or relatives were recruited nationwide through self-help associations. Relatives were asked to fill out the survey on behalf of the affected person. Health professionals were recruited through the federal chamber of psychotherapists and the National Association of Statutory Health Insurance Physicians in Germany. Cities and districts of the German states were sorted nationwide by population. An equally weighted selection of larger cities (more than 100,000 inhabitants) and smaller cities (less than 100,000 inhabitants) per state was made. Additionally, health professionals were recruited through non-inpatient help centers, such as social psychiatric services or counseling centers.

### 2.4. Statistical Analysis

Survey data were analyzed using descriptive statistics. Ninety-seven patients and relatives confirmed a mild to moderate depression diagnosis for the patient, and 229 health professionals completed the survey and were included for the presentation of results. The analysis of complete questionnaires was carried out separately for patients and relatives and health professionals. IBM SPSS statistics software, (version 26.0) was used for descriptive statistics.

## 3. Results

The survey was conducted between July and September 2020. This study’s total number of subjects was 1638, including 710 patients or relatives and 928 health professionals. Ninety-seven patients and relatives and 229 health professionals completed the survey.

### 3.1. General Characteristics of Study Subjects

In total, 69% of the patients or relatives consisted of women and 27% of men. Most subjects (70%) were over 40 years old and had a university degree (36%). Furthermore, 80% of the participants indicated that they or a relative received outpatient treatment from a general practitioner or specialist. Only one person stated to have received a DHI as a treatment option.

Moreover, 72% of the health professionals consisted of women and 24% of men. Most of the health professionals were older than 40 years old. Health professionals worked predominantly as medical and psychological psychotherapists (40%), social workers (29%), or physicians (9%). Furthermore, 47% of the sample worked in outpatient care (Table 1).

### 3.2. Technical Experience

**Figure 1:**
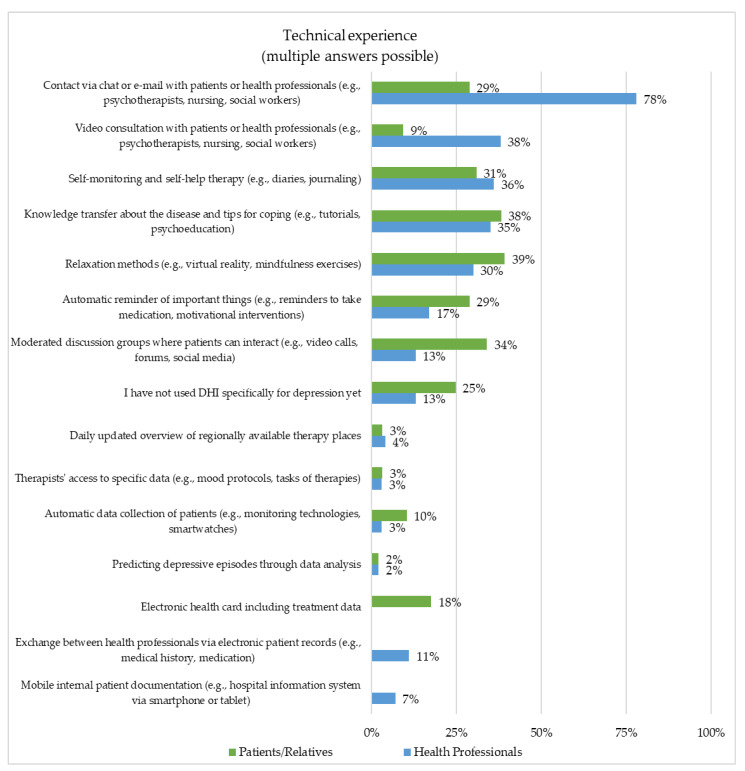
Representation of the technical experience (Patients/Relatives (*n* = 97) and Health Professionals (*n* = 229)).

In total, 87% of the patients or relatives and 84% of the health professionals indicated their technical experience to be positive. Technical experience is defined by previous professional and private experience with digital technology (e.g., internet, smartphone, tablet, PC, Alexa, Google Home). Most of the participants had previous experience with DHIs. Only 25% of the patients or relatives and 13% of the health professionals had not used a DHI for depression care yet. The subjects’ self-reported technical experience concerning DHI usage is presented in Figure 1.

### 3.3. Intention to Use

Approximately 80% of patients, relatives, and health professionals indicated being open to using a DHI for depression care. Only 14% of the patients or relatives and 13% of the health professionals stated not to be open to using DHIs.

### 3.4. Performance Expectancy

**Figure 2:**
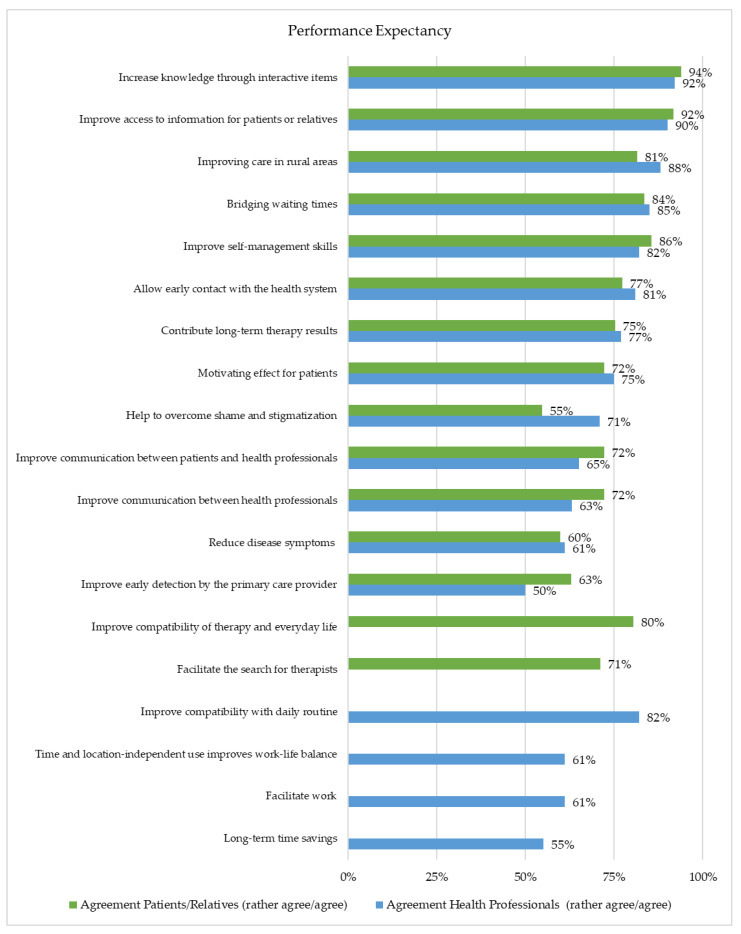
Representation of the Performance Expectancy (Patients/Relatives (*n* = 97) and Health Professionals (*n* = 229)).

Both groups’ highest agreement regarding performance-related attributions was for health literacy items. Over 90% agreed that access to information was improved for patients or relatives and that knowledge was increased through interactive items. The second- and third-highest levels of agreement were for bridging waiting times (patients or relatives: 84%; health professionals: 85%) and improving care in rural areas (patients or relatives: 81%; health professionals: 88%). Differences in the agreement were evident in the potential benefit of overcoming stigmatization. Here, 55% of the patients or relatives and 71% of the health professionals agreed. The improvement of early detection by the primary care provider (e.g., general practitioner) was also assessed differently (patients or relatives: 63%; health professionals: 50%). Approximately 60% of the subjects agreed that symptoms could be reduced with the help of a DHI (Figure 2).

### 3.5. Effort Expectancy

The participants most frequently feared problems integrating a DHI into everyday life in critical situations. Moreover, 80% of the patients or relatives strongly agreed that a DHI might be stressful and overwhelming in acute and critical situations. Moreover, there was a concern that the use of a DHI might be discontinued in the case of missing results (74%) and that help in critical situations would be inadequate (72%). Further, health professionals agreed that using a DHI could be stressful for elderly people (77%) and DHIs were not accessible to all population groups (90%).

**Figure 3:**
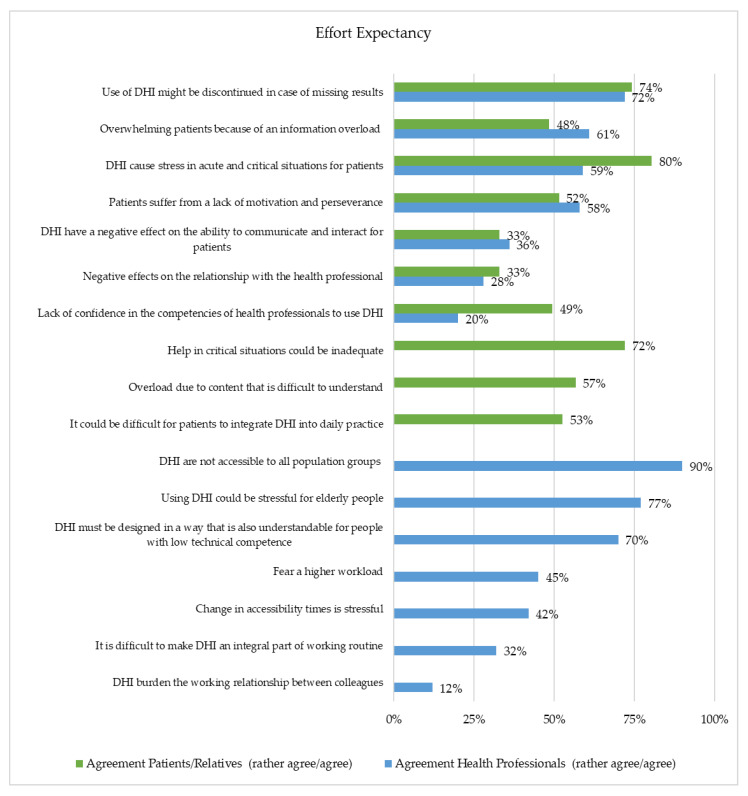
Representation of the Effort Expectancy (Patients/Relatives (*n* = 97) and Health Professionals (*n* = 229)).

Three items were assessed differently. First, there is a lack of confidence in the competencies of health professionals to use DHIs (patients or relatives: 49%; health professionals: 20%). In total, 53% of the patients or relatives agreed that it would be difficult for them to integrate a DHI into daily practice. Only 32% of the health professionals agreed that it would be difficult for health professionals to make a DHI an integral part of their working routine. Lastly, more health professionals (61%) than patients or relatives (48%) feared patients could be overwhelmed because of an information overload (Figure 3).

### 3.6. Facilitating Conditions

**Figure 4:**
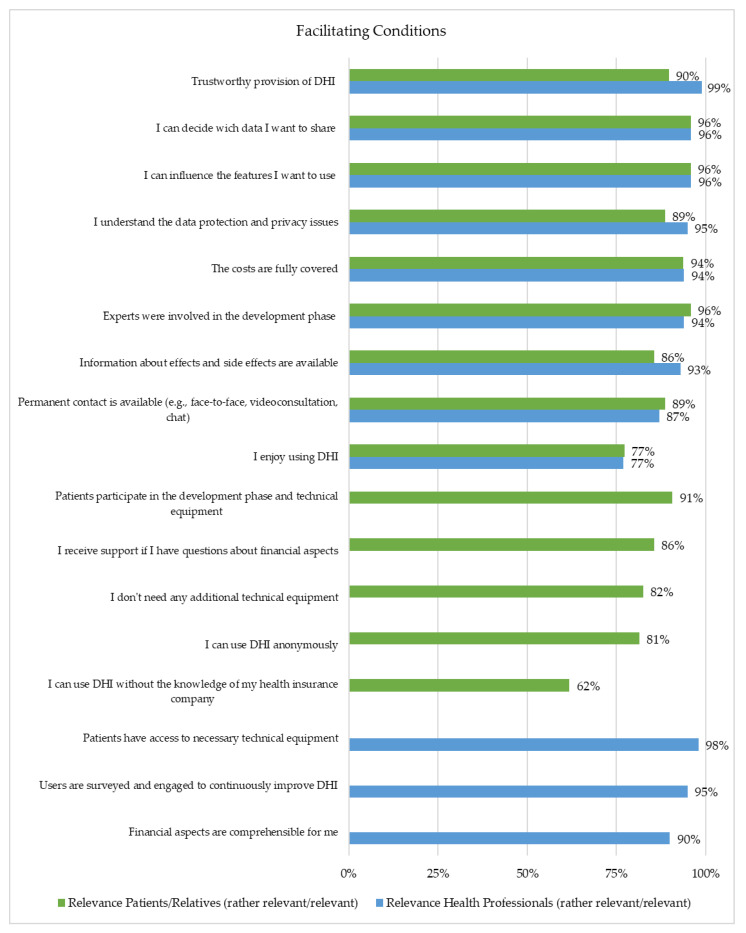
Representation of the Facilitating Conditions (Patients/Relatives (*n* = 97) and Health Professionals (*n* = 229)).

**Figure 5:**
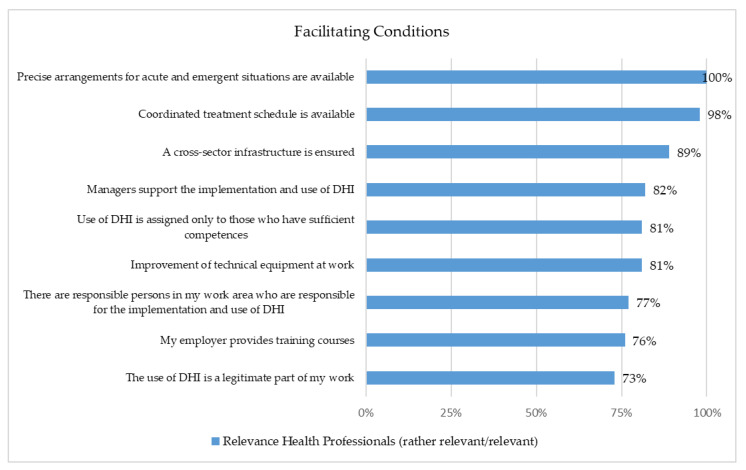
Representation of the Facilitating Conditions (Health Professionals (*n* = 229)).

Twenty-one of 26 items were assessed as important by at least 80% of the respondents (Figure 4 and Figure 5). These included data protection and privacy issues, financial aspects, participation in the development phase, and technical equipment. For 96% of the health professionals, as well as for 96% of the patients or relatives, it was important that they could influence the features they wanted to use and which kind of data they wanted to share. Furthermore, 96% of the patients or relatives and 94% of the health professionals stated that it is essential that experts were involved in the development phase (Figure 4).

From the perspective of health professionals, precise arrangements for acute and emergent situations (100%), trustworthy provision of DHI (99%), and a coordinated treatment schedule (98%) are the main aspects when using DHI for depression care (Figure 5).

### 3.7. Social Influence

**Figure 6:**
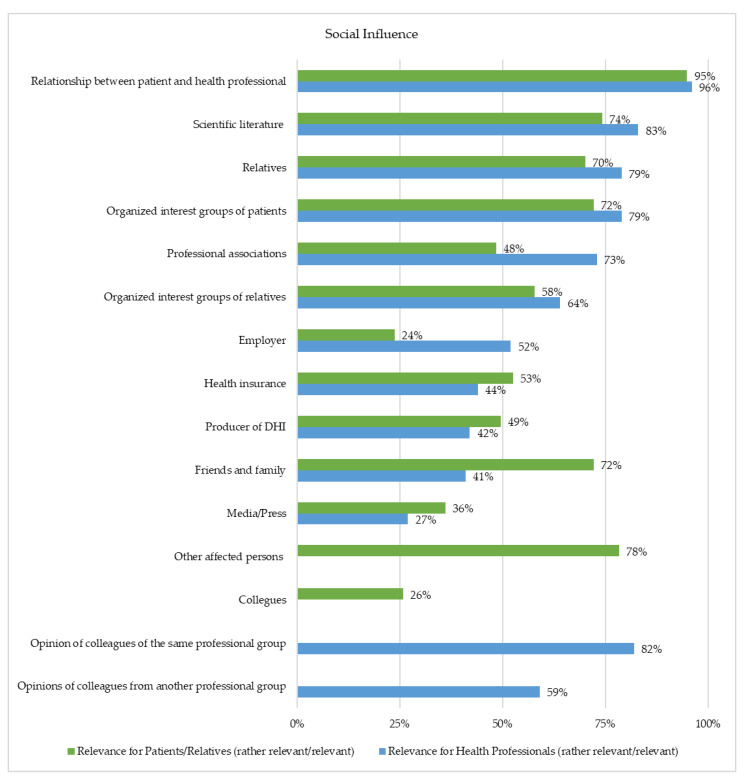
Representation of the Social Influence (Patients/Relatives (*n* = 97) and Health Professionals (*n* = 229)).

In the context of social influence, attitudes and opinions of essential others relevant to one’s own usage decision were assessed (Figure 6). Thus, 95% of patients or relatives and 96% of health professionals stated that the relationship between the patients and health professionals is important to them. Moreover, 74% of patients or relatives and 83% of health professionals indicated that scientific literature was meaningful. Furthermore, 78% of patients or relatives appraised other affected persons as the third most important source, whereas for 82% of the health professionals, the opinion of colleagues was the third most important.

## 4. Discussion

This survey is the first to report patients’ and their relatives’ and health professionals’ attitudes and acceptance towards DHIs for depression care in Germany. Over 80% of patients or relatives and 59% of health professionals described their subjective level of information as insufficient. This is in line with previous studies [14,16,20]. Consequently, transparent, understandable, and target-group-specific translations of scientific knowledge and the promotion of digital literacy are needed [10,16,19]. One study showed that people with less education, higher age, male gender, and low technology affinity have more straightforward access if they are actively informed [21]. Overall, access to target-group-specific information is necessary for health professionals and patients or relatives, as a powerful reciprocal influence can be assumed here. Especially for underserved and vulnerable groups, explicit invitations to low-barrier services are recommended [21].

The most important PE was aspects of health literacy and easy access to depression care. A recent survey shows that almost 50% of participants expect improvements in rural areas as a benefit of a DHI [10]. It is further confirmed that the use to improve self-management skills is supported by the majority [6,14,16,19,22]. The unclear approval concerning symptom improvement indicates that the respondents are rather unsure about this item. A meta-analysis of qualitative studies offers a possible explanation: Individual expectations affect the engagement of use. Accordingly, it could be conducive to use if expectations and preferences are clarified before actual use, thus preventing misunderstandings and the individual degree of support might be adjusted [23]. The groups assessed the item regarding overcoming stigmatization very differently. Health professionals may overestimate the effect since they mostly interact with patients who have sought help and have less contact with people who do not seek help. The two most central EEs of patients or relatives and health professionals were the discontinuation of use and insufficient help in acute phases. Both of these issues are also discussed in previous studies as the main reason for not using DHIs for mental health [24]. Moreover, 90% of the health professionals agreed that DHIs were not accessible for all affected people. This highlights the need for support services where technology literacy and access to technology are not conditions of use. Surprisingly, both groups assess the use of DHI in acute phases as overwhelming for patients differently (patients or relatives: 80%; health professionals: 59%). Possibly, the health professionals had a very treatment-related perception, lacking experience with possible excessive demands in the home environment. To identify overwhelming conditions in the home environment as early as possible, stress testing, as used in inpatient care, and access to direct care services might help.

The facilitating factors assessed as necessary by more than 80% were also discussed in previous studies: Low user-friendliness, lack of user-oriented design, insufficient privacy, DHIs as an untrustworthy source of mental health information, and insufficient usefulness for critical situations [24]. In particular, side effects and contraindications have been rudimentarily researched [6,25]. Meta-analyses found that the proportion of patients with clinically significant deterioration was not higher in intervention groups than in control groups, but publication bias cannot be excluded, and reasons for drop-out could not be considered [7]. It needs to be clarified which data requirements and legal frameworks are necessary [16,24,26]. In this context, the participation of experts and potential users in the development process and the transparent communication of information appear to be beneficial [14,16,27,28].

Furthermore, the results show that skill training for people involved in treatment, such as health professionals, patients, and their relatives, is crucial for the long-term acceptance of DHI. Training content should be adaptable to prior technology experience and affinity for technology. Although DHIs might increase the accessibility of treatment, their use still requires user engagement and effort. Current DHIs involve the attention and motivation of users and especially patients, which are unfortunately also characteristics that are in short supply for people who suffer from depression and could benefit most from treatment [29]. A previous systematic review, which discussed barriers and facilitators of DHIs, stated that a user´s digital health literacy influences the extent to which they are able to adapt and engage with DHI [13]. A population with low levels of digital literacy therefore requires novice-friendly interventions. Consequently, the improvement in digital literacy and technological skills might lead to higher motivation and engagement concerning DHI use. Additionally, the role of health professionals might be transformed due to technological development and the use of DHIs for depression treatment. As this survey showed, for most health professionals, it is important that the employer provides training courses handling DHI. Successful training must be supplemented with the direct observation of care delivery in real-world settings, and opportunities to practice these skills and receive feedback from peers and more experienced providers [30,31]. The most significant SI on usage decisions was the relationship between health professionals and patients. A review compared user acceptance of guided and unguided DHIs and reported heterogeneous study results [32]. A review of qualitative studies summarized that individual support was a major reason for using DHIs from users´ perspectives [23]. It is possible that for specific patient groups, personal contact is crucial for longer-term motivation to use the DHI [16,19,28,33]. Efficacy analyses showed that professional guidance of DHI is associated with a greater reduction in depression symptoms than unguided use [7]. Further research is needed to investigate the relevance of individual guidance for acceptance.

One of the main strengths of this survey was the multisided sample of participants representing the current status in Germany in terms of depression care. It is one of the few to examine the acceptance of patients with mild to moderate depression, their relatives and various health professionals. A major limitation is the question about diagnosis based on self-reports. It was not specified whether this was performed by doctors or psychotherapists. It cannot be ruled out that respondents also affirmed the diagnosis in the case of self-diagnoses or diagnoses by persons not qualified to do (e.g., non-medical practitioners). The validity of the questionnaire has not been statistically tested. However, international literature was involved, and cognitive pre-tests (*n* = 8) were conducted to assess the comprehensibility of the items and response options. Although the self-reported level of information of the participants regarding DHI was predominantly poor, it was possible to ensure that all respondents referred to this in their assessments by using a definition at the beginning of the survey. 

The survey was online, therefore the results could have been influenced by self-selecting bias. It can be assumed that the results are not transferable to patients, relatives, and health professionals who are more critical of technological developments, more reluctant to enter data via online media, and/or have lower technology skills. A previous study showed a very low willingness to participate among people who strictly reject the use of online media [19]. Furthermore, it is unclear whether the low response rate is related to a decreased motivation to use DHIs. Personal reasons (e.g., a lack of motivation, because of a medical condition) or circumstances of the research (e.g., availability of an incentive) could have been possible reasons for a low response rate. Depressive mood and a loss of energy and drive as characteristics of depressive disorders may also affect motivation. Moreover, research on user acceptance is vulnerable to selection bias because the process of accepting may already begin before using an innovative treatment. It is possible that people who have reservations regarding DHI do not participate in the first place [32].

Despite comprehensive recruiting strategies, the sample size was not large enough to generate representative conclusions. Additionally, most of the participants had experiences with DHI a priori, so a selection bias is not fully excluded. People who primarily use the Internet were reached. In Germany, this corresponds to approximately 91% of the population. Furthermore, 9% of the population are non-users [10]. The nationwide recruitment of patients or relatives through self-help associations may lead to overrepresentation. Persons with depression who do not use professional help services tended to be reached less. Since no data are available on relatives, it can be assumed that they are of a similar age and have a similar level of education as patients. Epidemiological data show that persons from lower educational groups are most frequently affected by depressive symptomatology in rather younger age groups [1]. In the present sample of patients and relatives, the majority are over 40 years old and have a university degree. Therefore, these groups are overrepresented. The representativeness is also limited because most of the participants were settled in North Rhine-Westphalia. This is a federal state that, to a large extent, is urbanized and so these factors have an impact on the appraisal of healthcare problems and the provision of healthcare services. Regarding the gender ratio of health professionals, the distribution is representative. In the sample of health professionals, the gender ratio (72% women) is nearly the same as the gender ratio of healthcare workers (75% women) [34].

In conclusion, it should be mentioned that the researchers decided against hypothesis testing for several reasons. First, the sample sizes are too small. In addition, the probability of random results increases due to the high number of hypotheses to be tested (problem of multiple comparisons). The goal is to avoid premature conclusions.

## 5. Conclusions

DHIs represent an innovative method to deliver depression treatment. Based on the results of this survey, some aspects of DHI acceptance and usage must be considered in the future. The most meaningful added value, and thus the highest acceptance of DHIs, is expected by bridging waiting times and providing access for affected persons in rural regions. The most common disadvantage is excessive demands in acute and critical phases and unequal access for vulnerable groups. This poses a risk of promoting inequalities in treatment provision due to differences in technical competencies and technical equipment. Many facilitating factors are essential for usage intention. One major challenge will be a participatory design in order to achieve acceptance. Additionally, access to reliable information is necessary for patients and health professionals, as reciprocal influence is to be assumed. The findings highlight the need to disseminate not only results of clinical and cost effectiveness but also to share best practices and clearly communicate for whom, when, and how digital treatments might be applied.

## Figures and Tables

**Table 1:** Characteristics of the sample.

	Patients/ Relatives (*n* = 97)	Health Professionals (*n* = 229)
**Gender**		
Female	69%	72%
Male	27%	24%
X-gender	2%	1%
Not specified	2%	3%
**Age**		
<20 years	0%	0%
20–30 years	13%	6%
31–40 years	15%	19%
41–50 years	20%	25%
51–60 years	30%	33%
>61 years	20%	16%
Not specified	2%	1%
**State**		
Baden-Württemberg	2%	7%
Bavaria	1%	4%
Berlin	3%	3%
Brandenburg	3%	5%
Bremen	0%	0%
Hamburg	4%	1%
Hesse	2%	7%
Mecklenburg-Western Pomerania	0%	3%
Lower Saxony	11%	8%
North Rhine-Westphalia	67%	45%
Rhineland-Palatinate	1%	2%
Saarland	0%	1%
Saxony	0%	4%
Saxony-Anhalt	4%	4%
Schleswig-Holstein	0%	4%
Thuringia	0%	2%
Not specified	2%	0%
**Degree of Urbanization**		
Small cities (<100,000 residents)	52%	39%
Large cities (>100,000 residents)	46%	61%
Not specified	2%	0%
**Highest Professional Degree**		
University degree	36%	69%
Technical college	7%	17%
Technical school	11%	8%
School-based training at a vocational college	23%	3%
Vocational training	14%	1%
No degree	2%	0%
No degree, but in vocational training	1%	0%
Other degree	0%	1%
Not specified	5%	1%
**Treatment Sector** (Multiple answers possible)		
Outpatient care	80%	47%
Inpatient care	30%	14%
Partial inpatient care	9%	7%
Social psychiatric service	7%	16%
Other service	8%	27%
No treatment	9%	-
Not specified	0%	0%
**Professional Group**		
Medical and psychological psychotherapists	-	40%
Social workers	-	29%
Specialist in neurology, specialist in psychiatry and psychotherapy	-	9%
Health care and nursing staff	-	10%
Occupational therapist	-	1%
Not specified	-	2%
Other professional group	-	9%

## Data Availability

The data presented in this study are available on request. The data are not publicly available due to privacy and ethical reasons.
